# Prevalent Pathogenic Variants of *ATP7B* in Chinese Patients with Wilson’s Disease: Geographical Distribution and Founder Effect

**DOI:** 10.3390/genes12030336

**Published:** 2021-02-25

**Authors:** Guo-Min Yang, Rou-Min Wang, Nan Xia, Zi-Wei Zheng, Yi Dong, Zhi-Ying Wu

**Affiliations:** Department of Neurology and Research Center of Neurology in Second Affiliated Hospital, and Key Laboratory of Medical Neurobiology of Zhejiang Province, Zhejiang University School of Medicine, Hangzhou 310009, China; guominyang@zju.edu.cn (G.-M.Y.); rouminwang@zju.edu.cn (R.-M.W.); xngrmuse@zju.edu.cn (N.X.); ziweizheng@zju.edu.cn (Z.-W.Z.); dongyi720@zju.edu.cn (Y.D.)

**Keywords:** Wilson’s disease, *ATP7B*, haplotype analysis, founder effect, Chinese

## Abstract

Wilson’s disease (WD) is an autosomal recessive disorder caused by *ATP7B* pathogenic variants. This study aimed to show the geographical distribution and haplotype spectrum of three prevalent pathogenic variants (p.R778L, p.P992L, p.T935M) in mainland Chinese population and clarify whether the founder effect may account for their origins. We firstly summarized the frequency and geographical distribution of p.R778L, p.P992L and p.T935M in 715 WD patients. Then, to construct haplotypes associated with the three variants, Sanger sequencing and microsatellite typing at three dinucleotide-repeat markers (D13S314, D13S301, D13S316) flanking the *ATP7B* gene were performed in 102 WD families. An obvious regional-specific distribution feature was found in p.T935M. Linkage disequilibrium at the three markers was shown in all the three variants and we found the common haplotypes specific for p.R778L, p.P992L and p.T935M respectively, represented successively by 10-7-7, 10-9-5 and 12-4-8, which all exhibited great significance vs. the control chromosomes (*p* < 0.01). Meanwhile, haplotypes for the three variants differed from the studies in other regions to some extent. The common haplotypes we found indicate that three prevalent pathogenic variants emerge due to the founder effect. Furthermore, the study contributes to expand our knowledge of the genetic diversity of WD from a cross-regional perspective.

## 1. Introduction

Wilson’s disease (WD), an autosomal recessive disorder, is characterized by abnormal copper metabolism resulting in the damage of various organs, predominantly hepatic and neurological impairment [1]. The diagnosis of the disease can be based on the typical clinical symptoms and corresponding biochemical findings such as reduced serum ceruloplasmin concentrations and elevated urinary copper excretion [2,3]. It has a prevalence rate of around 1/30,000 to 1/10,000 as well as a carrier frequency of about 1 in 90 among most populations [4,5], and it is believed that the prevalence is higher in China [6].

In 1993, the *ATP7B* gene accounting for the disease was identified [7,8,9]. At the same time, several highly polymorphic short tandem repeats (STRs) spanning the WD locus were found. These microsatellite markers were previously applied in the genetic linkage analysis of Chinese WD patients, which showed great contribution to the molecular diagnosis of potential carriers and asymptomatic patients [10]. In addition, Thomas et al. used the markers to explore the haplotype-variant associations [11,12], which were beneficial for explaining the origins of different variants. In this way, with recurrent pathogenic variants gradually identified in European populations, some scholars speculated about the origin from a common ancestor and then unveiled the possible founder effect for them [13,14,15]. For instance, one study comprised of Hungarian patients discovered the common haplotype for their most frequent variant p.H1069Q in a large proportion of subjects, pointing its origin from somewhere in eastern Europe [15].

Investigations of founder variants can help us trace the origins of variants, the evolution of the disease as well as the migration and growth of human populations [16], and there indeed exist different circumstances about common variants and population cultures of WD between Europe and Asia. Studies have previously summarized the geographical distribution of *ATP7B* in diverse populations in the world and found the higher prevalence of specific variants in certain populations, such as p.H1069Q in Europe and p.R778L in far east Asian countries [17,18]. Therefore, several haplotype studies in Asia were also performed to dig out the characteristics of their own common variants. In India, researchers recently considered the impact of traditional marriages in the same caste, and then detected the underlying founder effect for 14 WD recurrent variants including p.C271* and p.G711W [19]. Studies in other regions including Japan, China and Korea also conducted the haplotype analysis of *ATP7B* variants before [20,21,22,23], however, due to their target towards the whole variant spectrum, the number of their samples concerning frequent pathogenic variants such as p.R778L seemed inadequate, making the conclusion not so convincing. Meanwhile, there remained frequent differences among the variants and haplotype results of their studies.

Therefore, aiming to better decipher the genetic information about the potential founder effect of prevalent *ATP7B* variants, we utilized the p.R778L, p.P992L and p.T935M, the three most common pathogenic variants in the Chinese WD patients [24], to acquire their geographical distribution and analyze the haplotype-variant correlation with relatively large samples in a statistical way.

## 2. Materials and Methods

### 2.1. Subjects

To analyze the geographical distribution of three prevalent pathogenic variants, we recruited 715 WD patients reported in our previous study [3]. Then, 102 unrelated WD families were recruited for the current haplotype study. These families consisted of patients who had been identified with one or two of the three common variants by next-generation sequencing (NGS) and their unaffected relatives. They were enrolled between June, 2015, and October, 2020, in the Second Affiliated Hospital of Zhejiang University School of Medicine. All subjects originated from Chinese mainland. WD patients were clinically diagnosed according to the Leipzig Score [2]. This study was approved by the Ethics Committee of the Second Affiliated Hospital of Zhejiang University School of Medicine. Participants or their guardians provided informed consents.

### 2.2. Genotype Analysis

Genomic DNA was extracted using Blood Genomic Extraction Kit (Qiagen, Hilden, Germany) from peripheral EDTA-treated blood. The *ATP7B* variants of patients and their relatives were verified through Sanger sequencing, with a procedure described in our previous report [24]. For patients who were detected with only one heterozygous pathogenic variant, we performed multiplex ligation-dependent probe amplification assay (MLPA) with the *ATP7B* MLPA kit (SALSA P098-D1, MRC-Holland, the Netherlands) [25].

### 2.3. Haplotype Analysis

To derive the haplotype on each WD or normal chromosome, we used three microsatellite markers (D13S314, D13S301, D13S316) flanking the WD locus, which had previously been used for linkage analysis [10]. Specific primers for the amplification of these markers were described in previous studies [11,12], and one of each pair was labeled with fluorescent dye. The PCR was carried out in 10 μL total volume containing 50 ng genomic DNA, 1 mM of each primer and 7 μL KAPA 2G Robust Mix (KAPA Biosystems, Boston, MA, USA). The thermal condition was adjusted according to the previous study [22]. The PCR products were then quantified with deionized water. The mix including 4.2 μL Genescan 550HD size standard (Applied Biosystems, Foster City, CA, USA) with highly deionized formamide and 0.8 μL diluted PCR products was denatured at 95 °C for 5 min and chilled quickly to 4 °C. All the samples underwent the electrophoresis on the ABI Prism 3730 genetic analyzer (Applied Biosystems, Foster City, CA, USA) and the data were handled using the GeneMarke software (Applied Biosystems, Foster City, CA, USA). The size measurement was repeated three times independently for each sample.

### 2.4. Statistical Analysis

The geographical and allele distribution as well as haplotype association of three prevalent pathogenic variants were analyzed by chi-square test, with a Bonferroni correction or Fisher’s exact test when appropriate. WD chromosomes with the three variants were compared with normal chromosomes from the probands’ unaffected family members, as the controls. The analysis was performed in SPSS 20.0 (IBM Corp., Armonk, NY, USA). *p* value < 0.05 was regarded as statistically significant.

## 3. Results

### 3.1. Geographical Distribution of Three Prevalent Pathogenic Variants

**Figure 1:**
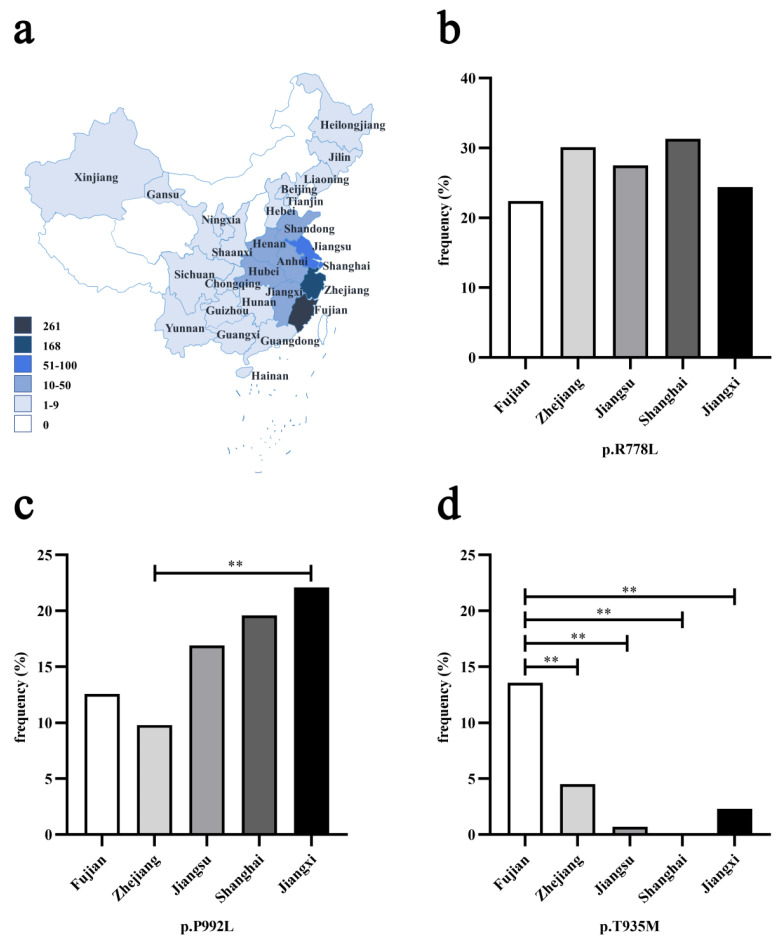
Geographical distribution of three *ATP7B* prevalent pathogenic variants (p.R778L, p.P992L, p.T935M) in Chinese mainland: (**a**) the geographical distribution of 709 Wilson’s disease (WD) patients. Various colors in different regions indicate the corresponding number range of patients; (**b**–**d**) the frequency of p.R778L, p.P992L and p.T935M in five regions (Fujian, Zhejiang, Jiangsu, Shanghai and Jiangxi). Five regions are represented by different colors. Asterisks indicate that there existed significant differences between the corresponding two provinces (** *p* < 0.01).

Among 715 unrelated WD patients, p.R778L, p.P992L and p.T935M were the three most prevalent pathogenic variants. Their allelic frequency was 31.7, 15.7 and 6.6%, respectively, which was close to what we reported previously [24]. Among 709 patients with geographical information, as shown in Figure 1a, the majority (579, 81.7%) were from southeastern China. Five regions accounted for the main proportion, in the order of Fujian province (261, 36.8%), Zhejiang (168, 23.7%), Jiangsu (71, 10.0%), Shanghai (56, 7.9%) and Jiangxi (43, 6.1%). Then, we compared the allelic frequency of three prevalent variants in these regions. There was no obvious difference in the allelic frequency of p.R778L among these regions (Figure 1b). For p.P992L, though its allelic frequency in Jiangxi (19/86, 22.1%) was significantly higher than that of Zhejiang (33/336, 9.8%) (*p* < 0.01), no other difference among the regions was seen (Figure 1c). However, as shown in Figure 1d, we found that the allelic frequency of p.T935M in Fujian (71/522, 13.6%) was significantly higher than that of Zhejiang (15/336, 4.5%), Jiangsu (1/142, 0.7%), Shanghai (0/112, 0.0%) or Jiangxi (2/86, 2.3%) (*p* < 0.01), impressively indicating the tendency for aggregation.

### 3.2. Linkage Disequilibrium at Three Markers for Three Prevalent Pathogenic Variants

Among 102 WD patients with the three prevalent pathogenic variants, biallelic variants were identified in each of them and successfully segregated in the corresponding relatives. There were 74 patients with c.2333G > T (p.R778L), 27 with c.2975C > T (p.P992L) and 13 with c.2804C > T (p.T935M). Among them, 26 patients were with homozygous p.R778L, two with homozygous p.P992L, one with homozygous p.T935M, eight with p.R778L and p.P992L, and four with p.R778L and p.T935M. The rest were all compound heterozygotes with one of the three prevalent variants and other variants. 

**Figure 2:**
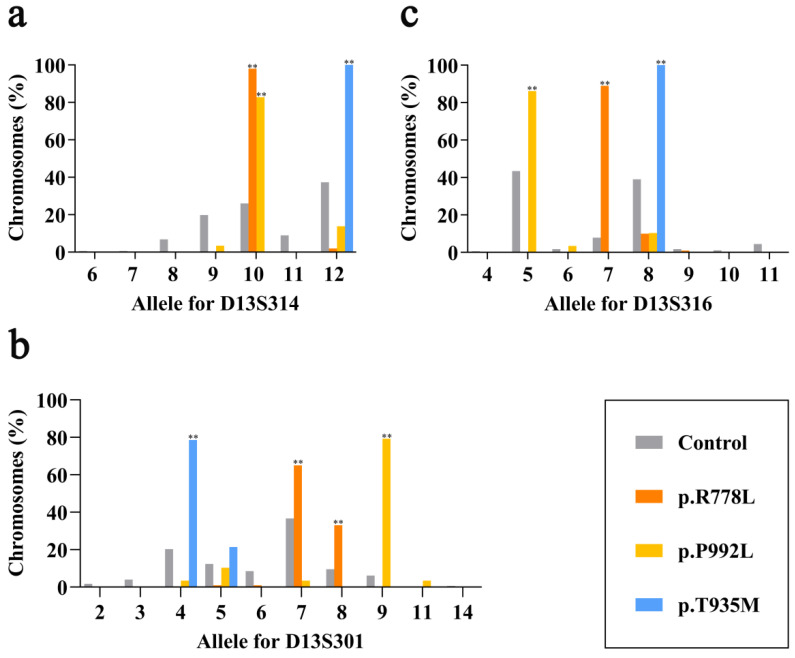
(**a**–**c**) Allele distribution of p.R778L, p.P992L and p.T935M at each of the three microsatellite markers (D13S314, D13S301, D13S316) in Wilson’s disease (WD) patients from mainland China. Brown boxes indicate normal chromosomes as the controls. Orange boxes indicate chromosomes with p.R778L, yellow boxes indicate chromosomes with p.P992L, and blue boxes indicate chromosomes with p.T935M. Asterisks indicate the frequency of chromosomes with the corresponding variant was significantly higher than that of control chromosomes in the same allele group (** *p* < 0.01).

To clarify the association of each specific marker with the variants, we firstly observed the allele distribution of p.R778L, p.P992L and p.T935M at each marker. The allele size definition for the three microsatellite markers (D13S314, D13S301, D13S316) used was based on a previous study [21]. In total, there were 100 chromosomes with p.R778L, 29 with p.P992L, 14 with p.T935M and 177 normal chromosomes in this study. As shown in Figure 2a, D13S314 exhibited significant linkage disequilibrium at allele 10 for chromosomes with p.R778L (98.0%) and p.P992L (82.8%) vs. control ones (26.0%, *p* < 0.01), while entirely linked at allele 12 for p.T935M (100 vs. 37.3%, *p* < 0.01). In terms of D13S301, we found the significant association of p.R778L with both allele 7 (65.0 vs. 36.7%, *p* < 0.01) and allele 8 (33.0 vs. 9.6%, *p* < 0.01), p.P992L with allele 9 (79.3 vs. 6.2%, *p* < 0.01) and p.T935M with allele 4 (78.6 vs. 20.3%, *p* < 0.01), respectively (Figure 2b). As shown in Figure 2c, D13S316 exhibited significant linkage distribution at allele 7 for p.R778L (89.0 vs. 7.9%, *p* < 0.01), allele 5 for p.P992L (86.2 vs. 43.5%, *p* < 0.01) and allele 8 for p.T935M (100.0 vs. 39.0%, *p* < 0.01). These results revealed that all three prevalent pathogenic variants had great linkage disequilibrium at each microsatellite marker, which might contribute to the haplotype association.

### 3.3. Haplotype Association of Three Prevalent Pathogenic Variants

With the alleles acquired at three microsatellite markers, we constructed the haplotypes for three prevalent pathogenic variants in 102 patients and found haplotype–variant correlations (Table 1). In light of the possibility that the new allele could be obtained through slippage during DNA replication from generation to generation [26], haplotypes differing by no more than one repeat unit at a single marker were gathered to one group.

The most prevalent pathogenic variant in China, p.R778L, was found to associate with three haplogroups (A, B, C). Haplogroup A was overwhelmingly common on WD chromosomes (97/100, 97.0%) vs. control ones (17/177, 9.6%, *p* < 0.01). It could be subsequently subdivided into haplotype variants A1–A4 (10-7-7; 10-7-8; 10-8-7; 10-8-8) and variant A1 (10-7-7) accounted for the largest proportion (57.0%). Haplogroup B (10-7-9) and C (12-5-7; 12-6-7) were found to be relatively scarce (1.0 and 2.0%, respectively) when compared with haplogroup A, suggesting that haplogroup A could exactly represent almost all the genetic information of p.R778L. 

However, the haplotype spectrum of p.P992L was more complex, including six haplogroups (D, E, F, G, H, I). Among them, haplogroup E was more common on WD chromosomes (21/29, 72.4%) than control ones (6/177, 3.4%, *p* < 0.01). It contained two variants (E1, E2), among which variant E1 (10-9-5) occupied the main part (69.0%) and variant E2 (10-9-6) was only found on one WD chromosome (3.4%). Haplogroup D (9-7-5), F (10-9-8), G (10-11-5), H (12-4-5; 12-5-5) and I (12-5-8) appeared sporadically, accounting for 3.4, 6.9, 3.4, 10.3 and 3.4% on WD chromosomes, respectively. Contrary to p.P992L, p.T935M presented a uniform state. There was only one kind of haplogroup on WD chromosomes, haplogroup J, which consisted of two variants J1 (12-4-8) and J2 (12-5-8), showing great significance vs. control ones (100 vs. 15.3%, *p* < 0.01). Additionally, variant J1 (12-4-8) was more common (78.6%).

## 4. Discussion

The origin of prevalent *ATP7B* pathogenic variants in Chinese WD population remains to be elucidated. Consequently, in this study, we first depicted the geographical distribution characteristics of three common variants (p.R778L, p.P992L, p.T935M) using a large WD cohort and then provided the haplotype spectrum of the three variants with a maximum sample size to date.

According to previous studies, the prevalent *ATP7B* pathogenic variants can vary by different populations in the world, and even show genetic heterogeneity in one certain country such as India, due to its ethnic diversity [17,18]. There also exist different constitutions of variants in the Chinese population of various districts. Though p.R778L was always detected as the most common pathogenic variant, the second common pathogenic variant was discovered to be p.A874V in one study of northern Chinese population [27] and p.I1148T primarily in the Guangdong province, Southern China [28], rather than p.P992L. All of these imply that *ATP7B* variants can have the characteristic of regional-specific distribution. As we discovered in this study, p.T935M was significantly associated with Fujian province, hinting at the possibility of a founder effect, while both p.R778L and p.P992L were not found to show such a specific tendency. These findings about the regional distribution of variants can help develop time-saving approaches and accelerate the genetic diagnosis of WD in specific regions, considering the vast diversity of the *ATP7B* variant spectrum.

We then observed in the haplotype spectrum that both p.R778L and p.T935M mainly had one haplogroup constituted by D13S314, D13S301 and D13S316, which could be represented by 10-7-7 and 12-4-8, respectively. Although there indeed existed some variants in the haplogroup, it could be noticed that there were only slight variations (no more than one repeat unit at a single locus), which could be explained by the allele slippage event [26]. This indicates the obvious founder effect of these two variants, especially p.T935M, which showed total single haplogroup and concentrated geographic distribution. For p.P992L, there were more kinds of haplogroups and their distribution seemed scattered, as haplogroups differed by more than one repeat unit at a single marker, even with the size interval over 10 bp. Despite this, we still found that haplogroup D, particularly variant D1, 10-9-5, accounted for the majority in the series. Therefore, such variations might not indicate independent origins of the same variant, but emerge as a result of multiple allele slippages or recombination events with other haplogroups on an old common ancestor during a very long period [12].

To compare our studies with others, in Table 2, we summarized the results involving the three prevalent variants in other areas including Japan, Taiwan, China and Hong Kong, China [20,21,22]. Both Japan and Taiwan, China, had two related studies, and we chose one with more samples, respectively. Consistent with our study, it was also rare that there was only one pure haplotype for each variant in those regions. Meanwhile, the haplotypes of three variants all showed discrepancies to an extent in comparison with their studies. Firstly, it was noticeable that the most apparent difference between Japanese and Chinese population lay in the marker D13S314. For Taiwan, China, when comparing the most common haplotype of p.R778L and p.P992L, we found that the allele at each marker in our study exceeded that of theirs by nearly 2–3 units. Furthermore, the data of the study from Hong Kong, China, could be the closest with ours, though there still remained variations, mainly in the marker D13S301. Apart from them, Korean scholars also studied the haplotype feature of p.R778L before [23], though the same marker they utilized was D13S316 alone and they used a different allele size definition. We also noticed the similar phenomenon that their main allele size for p.R778L at this marker was 164, which showed great distance from our result 142. The reasons accounting for the multiplicity among the studies could include the lack of consensus on allele size definition, different amplification primer sequences or approaches to the marker size measurement used and importantly, the heterogeneity which originally existed among different regions [21].

Unlike the diversity of haplotype pattern among Asian regions, it is noteworthy that the most prevalent *ATP7B* pathogenic variant for Europe, p.H1069Q, was found with the same haplotype in an overwhelming proportion of subjects from various European countries including Austria, Germany, and the United Kingdom [15]. This may indicate different circumstances about the migration and growth of human populations between the two continents, which could be explained by relevant history.

Actually, the other studies for the three variants all reflected certain tendencies of founder effect, however, the number of WD chromosomes carrying each variant might not be so adequate, which should constitute the strength of our study. On the other hand, our study also has some limitations. For instance, the method of capillary electrophoresis may induce tiny deviations in some families, which sometimes cannot uncover the most precise size of the marker. In addition, first-degree siblings of probands were not recruited in this study to strengthen the evidence. Furthermore, to better understand the founder effect in WD, other frequent pathogenic variants in China might also need further distinct investigation.

## 5. Conclusions

In summary, our study accomplished the first analysis about geographical distribution and haplotype spectrum with large samples for three prevalent pathogenic variants of *ATP7B* encompassing p.R778L, p.P992L and p.T935M in the Chinese mainland. We showed that p.T935M had a tendency of regional-specific distribution and all of three variants could possess underlying founder effect for their inheritance and the haplotypes varied from one region to another to some extent. The results facilitate the explanation for the origins of *ATP7B* variants with high frequency in China as well as provide a better knowledge of the genetic diversity of WD from a cross-regional perspective. 

## Figures and Tables

**Table 1:** Haplotypes with D13S314, D13S301, D13S316 for three prevalent pathogenic variants in Wilson’ s disease (WD) patients from mainland China.

Variant	Group ^1^		Haplotype			No. of Chromosomes (%)	
			D13S314	D13S301	D13S316	WD	Control (*n* = 177)
p.R778L (*n* = 100)	A	A1	10	7	7	57(57.0)	4(2.3)
		A2	10	7	8	7(7.0)	3(1.7)
		A3	10	8	7	30(30.0)	8(4.5)
		A4	10	8	8	3(3.0)	2(1.1)
	Subtotal					97(97.0) **	17(9.6)
	B		10	7	9	1(1.0)	0(0.0)
	C	C1	12	5	7	1(1.0)	0(0.0)
		C2	12	6	7	1(1.0)	0(0.0)
p.P992L (*n* = 29)	D		9	7	5	1(3.4)	13(7.3)
	E	E1	10	9	5	20(69.0)	6(3.4)
		E2	10	9	6	1(3.4)	0(0.0)
	Subtotal					21(72.4) **	6(3.4)
	F		10	9	8	2(6.9)	0(0.0)
	G		10	11	5	1(3.4)	0(0.0)
	H	H1	12	4	5	1(3.4)	5(2.8)
		H2	12	5	5	2(6.9)	8(4.5)
	I		12	5	8	1(3.4)	9(5.1)
p.T935M (*n* = 14)	J	J1	12	4	8	11(78.6)	18(10.2)
		J2	12	5	8	3(21.4)	9(5.1)
	Subtotal					14(100.0) **	27(15.3)

“^1^” represent different haplogroups are represented by corresponding letters. The haplotype variants in the same haplogroup are displayed by the same letter with Arabic numbers in order. Asterisks indicate the frequency of chromosomes with the corresponding variant in this haplogroup was significantly higher than that of control chromosomes (** *p* < 0.01).

**Table 2:** Haplotypes with D13S314, D13S301, D13S316 for three prevalent pathogenic variants in Wilson’s disease (WD) patients from Japan, Taiwan, China and Hong Kong, China.

Study	Variant	Haplotype			No. of Chromosomes
		D13S314	D13S301	D13S316	
Japan [20]	p.R778L	5	5	6	1
		5	7	4	2
		5	7	5	1
		5	7	5.5	4
		5	7	7	1
	p.P992L	6	9	4	3
Taiwan, China [21]	p.R778L	8	4	4	4
		8	4	5.5	4
	p.P992L	8.5	6.5	2	2
		8.5	6.5	5.5	3
Hong Kong, China [22]	p.R778L	10	4	7	5
		10	5	7	2
		10	6	7	5
		10	9	7	1
		13	3	7	1
	p.P992L	10	7	4	7
		10	7	8	1
	p.T935M	13	1	8	1
		13	3	8	1

## Data Availability

No new data were created or analyzed in this study. Data sharing is not applicable to this article.
